# Effect of dexmedetomidine on postoperative cognitive function in patients with gastrointestinal cancer: a meta-analysis of randomized controlled trials

**DOI:** 10.3389/fneur.2025.1605999

**Published:** 2025-08-21

**Authors:** Xiaoxia Zheng, Yang Lan, Lesi Chen, Ruiming Du, Jiaxuan Wu

**Affiliations:** ^1^Department of Anesthesia, The Second Affiliated Hospital of Shantou University Medical College, Shantou, Guangdong, China; ^2^Department of Anesthesia, Shenzhen TCM Anorectal Hospital (Futian), Shenzhen, Guangdong, China

**Keywords:** dexmedetomidine, gastrointestinal cancer, postoperative cognitive function, meta-analysis, mini-mental state examination

## Abstract

**Introduction:**

This meta-analysis was conducted to systematically evaluate the effects of dexmedetomidine (DEX) on mini-mental state examination (MMSE) scores and the incidence of postoperative cognitive dysfunction (POCD) in patients with gastrointestinal cancers (GICs) undergoing radical surgery (RS), by aggregating data from randomized controlled trials (RCTs).

**Methods:**

A comprehensive literature review was undertaken that encompassed seven databases from their inception until March 4, 2024. The quality of the studies was assessed using the Cochrane Collaboration tool to evaluate risk. Based on the heterogeneity determined through Cochran’s Q and I^2^ tests, either fixed-effect or random-effect models were employed to conduct the appropriate meta-analyses. Publication bias was assessed using the Egger test, while the stability of the results was evaluated through a one-by-one elimination method.

**Results:**

A total of 12 studies involving 881 patients with GIC (440 patients treated with DEX and 441 patients receiving saline) were included in this meta-analysis. The overall quality of the included studies was deemed moderate. The application of a random-effect model indicated that DEX significantly elevated MMSE scores on postoperative days 1, 2, 3, and 7, albeit with considerable heterogeneity. Conversely, the fixed-effect model demonstrated a protective effect of DEX on the incidence of POCD. Nonetheless, subgroup analyses stratified by cancer type and surgical method did not identify the sources of heterogeneity. The Egger test revealed no evidence of publication bias across the included studies (*p* = 0.447). Sensitivity analyses further confirmed the robustness of the findings of this meta-analysis.

**Discussion:**

The findings suggest that DEX exerts a protective effect on cognitive function in patients with GICs undergoing RS. Nevertheless, high-quality, large-scale RCTs are necessary to furnish more definitive evidence.

## 1 Introduction

Gastrointestinal cancers (GICs), including esophageal, gastric, colon, and rectal cancers (RC), account for approximately 20% of all cancer diagnoses worldwide (1). According to 2018 estimates, 36.4% of digestive system cancers in China have a very poor prognosis, with a very low 5-year overall survival rate (OS) (less than 35% between 2013 and 2015) (2). The poor prognosis of GICs is closely linked to systemic immune dysregulation. Previous studies have suggested that disease progression in GIC is accompanied by systemic immune disorders and functional changes in various immune cells, including T cells, neutrophils, macrophages, and monocytes (3, 4). While radical surgery (RS) offers a potential cure, the associated anesthesia may exacerbate neuroinflammation, contributing to postoperative cognitive dysfunction (POCD) (5–7). POCD is a frequent complication among elderly patients, characterized primarily by one or more cognitive impairments that occur post-surgery (8). POCD detrimentally affects surgical recovery, prolongs hospital stays, diminishes quality of life, and increases mortality risk (9). Therefore, a comprehensive assessment of the safety and efficacy of drugs aimed at improving POCD in patients with GICs is warranted.

Anesthetic drugs have a certain effect on the nervous system and are associated with clinical manifestations such as cognitive impairment and memory loss (10, 11). Even in short surgical procedures, exacerbated cognitive impairment can occur, posing a significant concern (12–14). Given these risks, pharmacological interventions like Dexmedetomidine (DEX), which modulates both inflammation and neuronal activity, have gained attention. DEX is an α2 adrenergic receptor agonist that acts on the CNS (15). Its sedative, analgesic, anxiolytic, and respiratory-protective properties have made it a widely used essential anesthetic drug during the perioperative period (16). DEX provides sedation and analgesia without respiratory depression, unlike midazolam or propofol, while also improving pain tolerance (17). Additionally, DEX has the potential to reduce perioperative stress and inflammation risks and reduce postoperative complications (18). Perioperative DEX is beneficial in reducing stress levels (19, 20), while postoperative intravenous use is effective in reducing inflammation and improving cognitive function in older adults (21). In addition, studies have shown that DEX may be associated with immune function protection in patients undergoing cancer surgery and potential inhibition of tumor cell growth (22). In the context of GICs, DEX has been linked to improvements in POCD, stabilization of cardiovascular hemodynamics, and possible cerebral neuron protection (23). Liao et al. (8) and Liu et al. (24) reported that DEX administration was accompanied by faster postoperative recovery and notable reductions in cognitive dysfunction in patient. In contrast, Ning and others found no significant distinction in the effects on cognitive function between DEX and saline at 7 days postoperatively (25). These observations reflect ongoing uncertainty regarding the presence of a protective effect of DEX against POCD in patients with GICs undergoing RS (20–22).

The mini-mental state examination (MMSE) is a composite tool frequently used to assess POCD, with approximately 21% of studies employing this method (26). Therefore, this meta-analysis systematically evaluates DEX versus saline effects on MMSE scores and POCD incidence in GICs patients undergoing RS, using pooled RCT data. These findings also provide valuable suggestions for clinical treatment and the improvement of patient prognosis.

## 2 Methods

### 2.1 Data retrieval strategy

This meta-analysis was registered in PROSPERO before its implementation (CRD42024523917). Literature was retrieved from PubMed, Embase, the Cochrane Library, Web of Science, Wanfang Data, the China National Knowledge Infrastructure, and the China Science and Technology Journal databases. The keywords “dexmedetomidine,” “precedex,” “neoplasms,” “cancer,” “cognition,” and “cognitive” were combined and searched by logical relationships of “OR and.” During this process, a pattern of integrating subject heading terms with free-text words was adopted, with search strategies adjusted according to the specific characteristics of the respective databases. The search details for the four English databases are presented in Supplementary Tables 1–4. To avoid selection bias, all literature published up until March 4, 2024 was searched, without imposing any language restrictions. Additionally, reference lists from relevant reviews and included studies were also consulted. While no language restrictions were applied, our search specifically included Chinese databases to capture regionally relevant studies.

### 2.2 Study selection criteria

Literature was deemed eligible for inclusion in this review if (1) the study was conducted on patients with surgically treated GIC (including gastric, colorectal, colon, and rectal cancers); (2) the study evaluated differences in the effects of DEX and saline on POCD; (3) the study was an RCT; and (4) the study reported one or more of the following outcomes: MMSE score, case number, or incidence of POCD.

Exclusion criteria were as follows: (1) non-original studies, including reviews, conference abstracts, and comments; (2) studies lacking inclusion criteria, did not report POCD criteria or measurement time, or had data errors; and (3) for duplicate publications or multiple articles sharing the same data, only the one with the most complete information was included.

### 2.3 Data extraction and quality assessment

Two investigators (Xiaoxia Zheng and Yang Lan) independently conducted literature screening based on the aforementioned criteria and extracted data according to a pre-specified data field. For each eligible article, information including the name of the first author, publication year, basic characteristics of the study subjects (such as sample size and age), type of cancer, type of surgery, method of anesthesia, and study outcomes were collected. Following the completion of data extraction, the fields were exchanged for review, and any inconsistencies were negotiated. The Cochrane Collaboration tool for assessing risk was employed for the quality assessment of the RCTs (27).

### 2.4 Outcome assessment instruments

The MMSE scale, a widely used 30-point scale assessing cognitive domains including orientation (10 points), registration (3 points), attention/calculation (5 points), recall (3 points), and language/visual construction (9 points), was utilized to evaluate cognitive dysfunction. In most of the included studies, POCD was defined as an MMSE score <27 at any time point after surgery. The diagnostic criteria and assessment time points for POCD in the included studies are detailed in Supplementary Table 5.

### 2.5 Statistical analysis

Data analysis was performed using RevMan 5.3 and Stata12.0. This meta-analysis compared the differences in MMSE scores and the incidence of POCD at different follow-up times (1, 2, 3, and 7 days postoperatively). Among the differences in POCD incidence, the risk ratio (RR) and 95% confidence interval (CI) were used as effect values. The MMSE score, as a continuous variable, was assessed using the weighted mean difference (WMD) and 95% CI as effect values. Heterogeneity across the studies was monitored using Cochran’s *Q* and *I*^2^ tests (28). The random-effects model was implemented if significant heterogeneity was defined at *p* < 0.05 or *I*^2^ > 50% (*I*^2^ statistic); otherwise, the fixed-effects model was adopted. A subgroup analysis was conducted to estimate the effect of cancer and surgery type on heterogeneity and the pooled effect. Egger’s test and the one-by-one elimination method were used to monitor publication bias across studies and the stability of pooled estimates, respectively (29, 30).

## 3 Results

### 3.1 Literature retrieval

**Figure 1:**
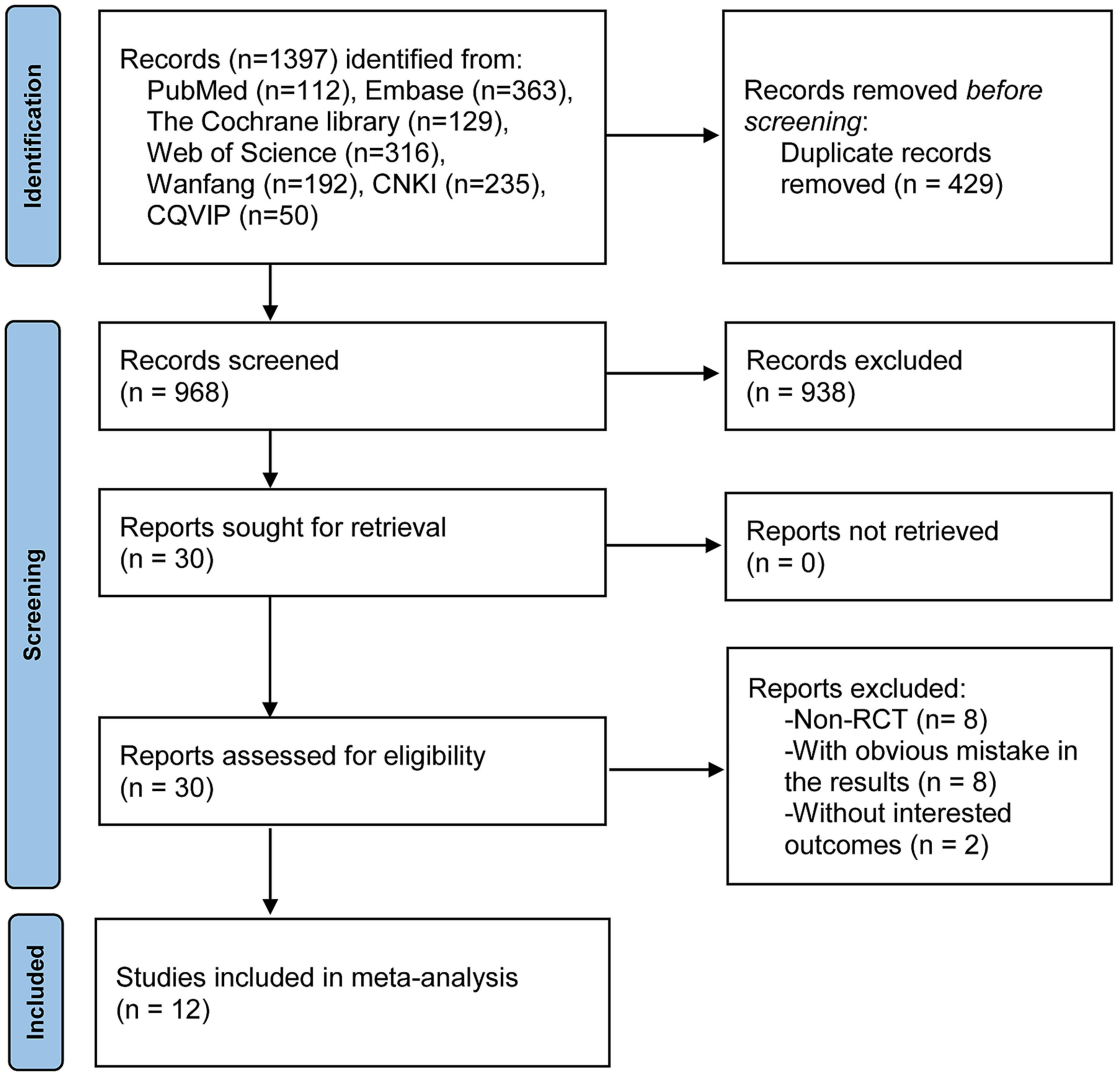
Illustration of the process and results of literature retrieval.

The process and results of the literature search are shown in Figure 1. A total of 1,397 records were obtained from online databases. After an initial screening by browsing the titles and abstracts, 429 duplicates and 938 articles that did not meet the inclusion criteria were excluded. Subsequently, after a thorough reading of the full text and screening of the remaining 30 articles, 12 studies were finally included in this meta-analysis (8, 24, 25, 31–39).

### 3.2 Characteristics and quality assessment of included studies

As shown in Table 1, all the 12 RCTs included in this meta-analysis were conducted in China. Among these, five studies were conducted on patients with gastric cancer (GC) (8, 33, 36, 37, 39), six on patients with rectal cancer (RC), including colorectal cancer (CRC) (24, 25, 31, 32, 35, 38); and one on patients with GIC (34). Regarding the type of surgery, seven publications included subjects undergoing laparoscopic RS (LRS) (8, 25, 32, 33, 35, 37, 39), whereas the remaining five reported only RS (24, 31, 34, 37, 38). The sample size of the 12 studies ranged from 60 to 120, with 881 subjects (440 in the DEX and 441 in the saline groups) enrolled in this meta-analysis. In all included studies, there were no significant differences in age, sex, or American Society of Anesthesiologists classification between the DEX and saline groups.

**Table 1:** Characteristics of 12 included studies in this meta-analysis.

Study	Patients	Surgery	ASA	Anesthesia method	Use of DEX	Groups	*n*, M/F	Age, years
Geng et al. (33)	GC	LRS	II–III	I.V	Bolus (0.5 μg/kg) before induction, continuous infusion (0.1 μg/kg/h) during operation	DEX	30, 17/13	66.58 ± 3.17
						Saline	30, 16/14	66.86 ± 3.29
Liao et al. (8)	GC	LRS	II–III	I.V	Bolus (0.5 μg/kg) before induction, continuous infusion (0.3–0.5 μg/kg/h) during operation	DEX	35, 20/15	71.26 ± 3.58
						Saline	35, 21/14	69.69 ± 2.52
Liu and Xie (34)	GIC	RS	I–III	I.V	Bolus (0.5 μg/kg) before induction, continuous infusion (0.3 μg/kg/h) during operation	DEX	30, 16/14	68 ± 9
						Saline	30, 18/12	67 ± 8
Liu et al. (35)	CRC	LRS	I–III	I.V	Bolus (1.5 μg/kg) before induction	DEX	48, 25/23	60–76
						Saline	48, 26/22	60–78
Liu et al. (24)	CRC	RS	I–II	I.V	Bolus (0.5 μg/kg) before induction, continuous infusion (0.6 μg/kg/h) during operation	DEX	24, 15/9	69.6 ± 4.4
						Saline	24, 13/11	68.6 ± 3.9
Lu and Chen (31)	CRC	RS	I–III	I.V	Bolus (0.5 μg/kg) before induction, continuous infusion (0.25 μg/kg/h) during operation	DEX	30, 18/12	67.2 ± 4.1
						Saline	30, 15/15	68.4 ± 4.6
Ma et al. (39)	GC	LRS	II–III	Combine	Bolus (1.0 μg/kg) before induction, continuous infusion (0.5 μg/kg/h) during operation	DEX	30, 18/12	71.4 ± 3.1
						Saline	30, 16/14	72.5 ± 2.4
Ning and Xie (25)	RC	LRS	I–II	Combine	Bolus (0.5 μg/kg) before induction, continuous infusion (0.4 μg/kg/h) during operation	DEX	31, 16/15	60.05 ± 5.34
						Saline	31, 18/13	60.89 ± 5.90
Shi et al. (36)	GC	RS	I–II	I.V	Bolus (0.5 μg/kg) before induction, continuous infusion (0.1 μg/kg/h) during operation	DEX	35, 22/13	65.59 ± 2.71
						Saline	35, 23/12	65.61 ± 2.28
Zeng et al. (32)	RC	LRS	I–II	I.V	Bolus (1.0 μg/kg) before induction, continuous infusion (0.15 μg/kg/h) during operation	DEX	60, 39/21	62.9 ± 6.9
						Saline	60, 36/24	63.5 ± 7.8
Zhang et al. (37)	GC	LRS	NR	I.V	Bolus (1.0 μg/kg) before induction, continuous infusion (0.5 μg/kg/h) during operation	DEX	50, 31/19	69.6 ± 11.3
						Saline	50, 28/22	71.2 ± 12.4
Zheng et al. (38)	CRC	RS	I–III	I.V	Bolus (2.0 μg/kg) before induction, continuous infusion (0.2–0.5 μg/kg/h) during operation	DEX	37, 20/17	60.2 ± 7.1
						Saline	38, 21/17	60.2 ± 7.1

ASA, American Society of Anesthesiologists; DEX, dexmedetomidine; F, female; M, male; CRC, colorectal cancer; RC, rectal cancer; GC, gastric cancer; GIC, gastrointestinal cancer; I. V, intravenous injection; combine, inhaled + I.V; LRS, laparoscopic radical surgery; RS, radical surgery.

The results of the quality assessment are shown in Supplementary Figure 1. The included studies lacked details regarding blinding, and most of them did not report specific randomization and allocation concealment schemes. Therefore, these studies showed moderate risks of selection, performance, and detection biases. The risk of bias summary table is shown in Supplementary Table 6. The GRADE system was used to grade the quality of evidence, with assessment dimensions including study design, risk of bias, inconsistency, indirectness, imprecision, and publication bias. Due to the high risk of bias in the included studies (unclear randomization methods, inadequate blinding), significant heterogeneity (*I*^2^ > 50%), and the fact that all studies were from China (with some indirectness), the quality of evidence for MMSE scores 1–7 days post-surgery was rated as ‘low.’ For the incidence of POCD, although heterogeneity was low (*I*^2^ < 50%), there was still a high risk of bias (unclear blinding) and geographical limitations, and the quality of evidence was rated as ‘moderate.’

### 3.3 Meta-analysis

**Figure 2:**
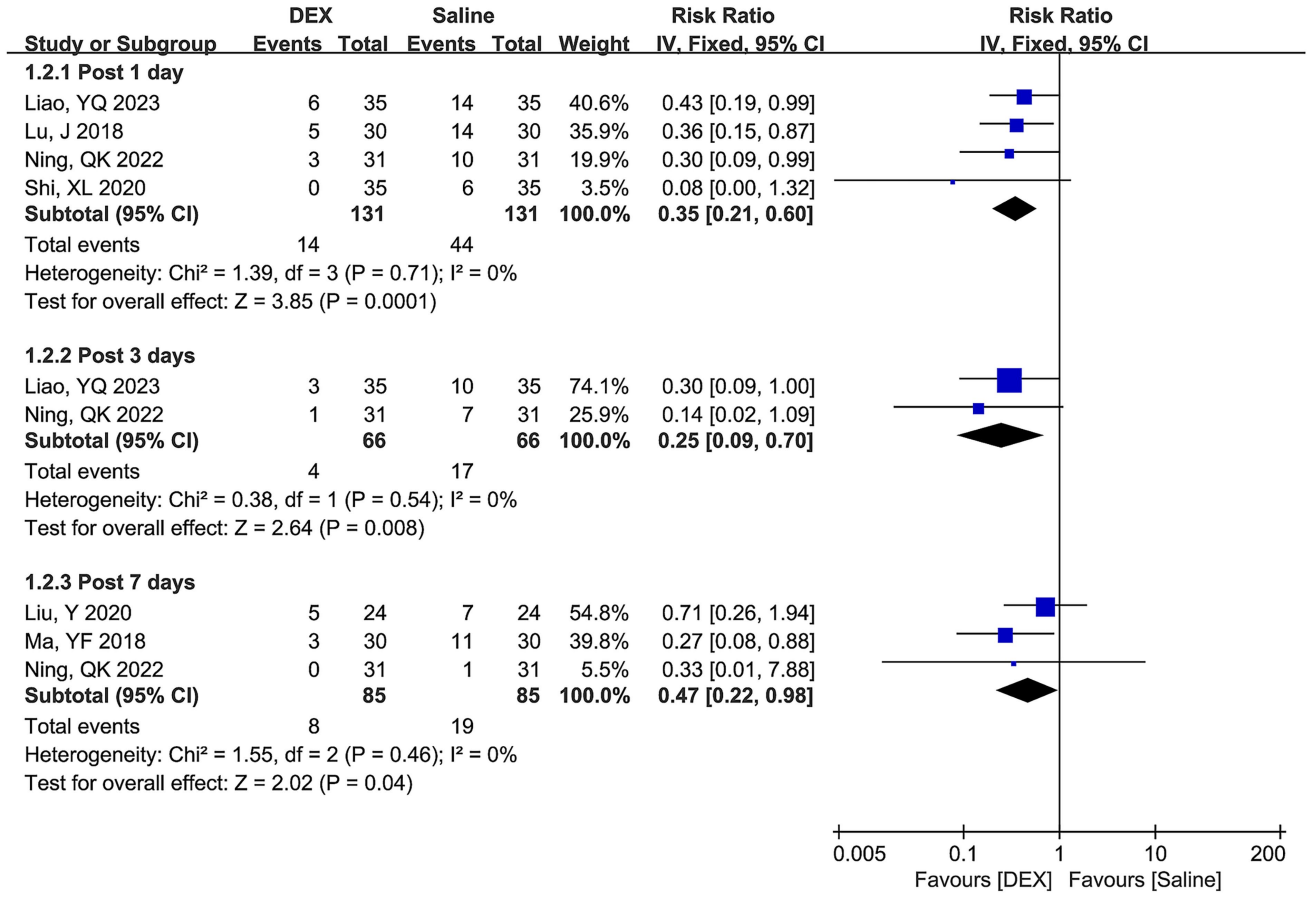
Forest plots depicting differences in MMSE score between the DEX and saline groups at postoperative days 1, 2, 3, and 7.

The differences in the MMSE scores between the DEX and saline groups on postoperative days 1, 2, 3, and 7 are depicted in Figure 2, representing significant heterogeneity among the included studies across all the time points. The pooled results of the random-effect model suggested that DEX significantly increased the MMSE score on postoperative days 1 (WMD [95% CI] = 2.53 [1.88, 3.19], *p* < 0.00001), 2 (WMD [95% CI] = 2.53 [1.07, 3.99], *p* = 0.0007), 3 (WMD [95% CI] = 2.24 [0.97, 3.51], *p* = 0.0006), and 7 (WMD [95% CI] = 1.82 [0.73, 2.92], *p* = 0.001).

**Figure 3:**
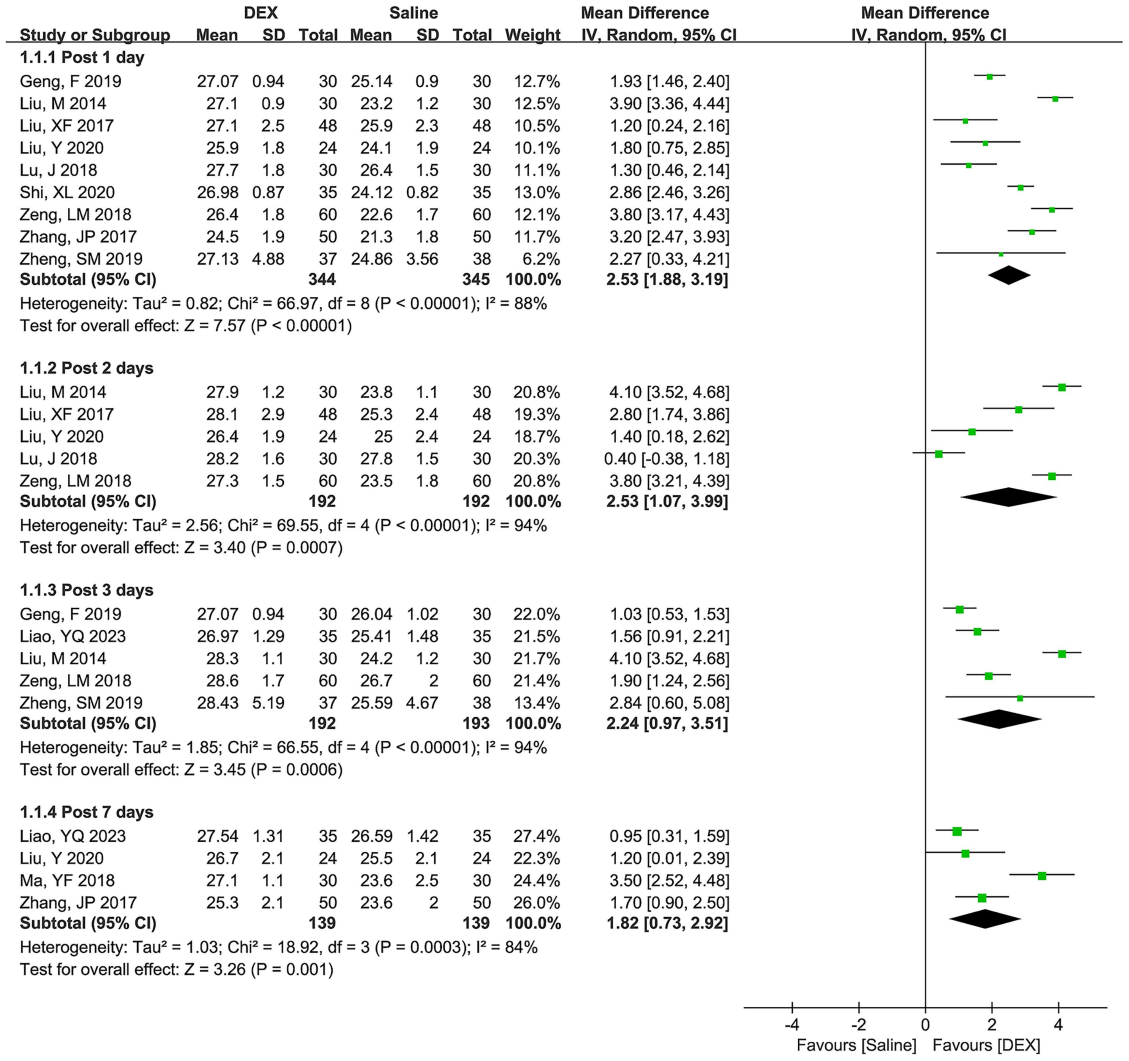
Forest plots depicting differences in POCD incidence between the DEX and saline groups at postoperative days 1, 3, and 7.

Figure 3 shows the differences in POCD incidence between the two groups at postoperative days 1, 3, and 7. No significant heterogeneity was observed across all time points, and the combined estimates of the fixed-effect model indicated that DEX significantly decreased the risk of POCD incidence compared with the use of saline at postoperative days 1 (RR [95% CI] = 0.35 [0.21, 0.60], *p* = 0.0001), 3 (RR [95% CI] = 0.25 [0.09, 0.70], *p* = 0.008), and 7 (RR [95% CI] = 0.47 [0.22, 0.98], *p* = 0.04).

### 3.4 Subgroup analysis and publication bias test

**Figure 4:**
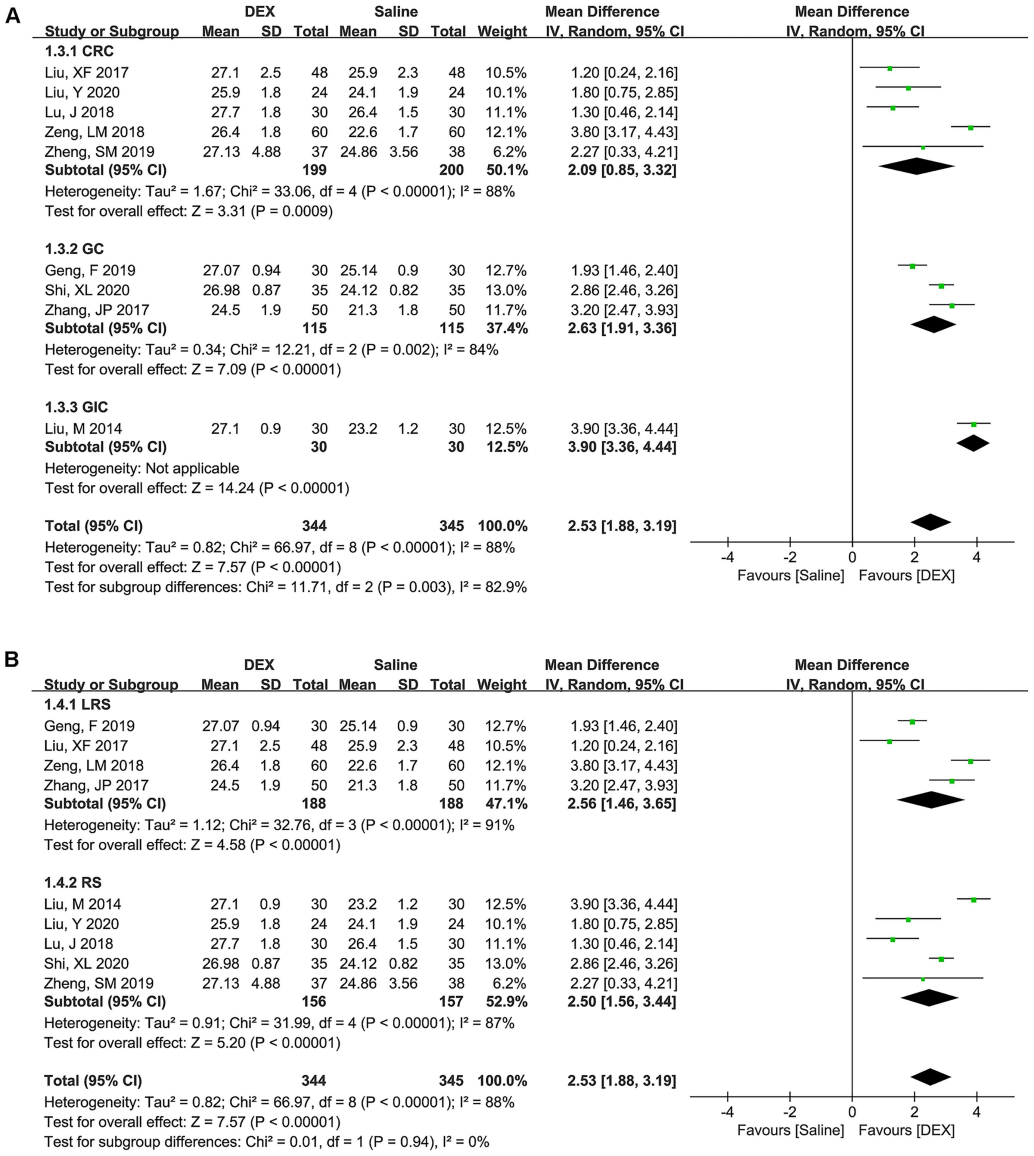
Subgroup analysis based on cancer type **(A)** and surgery type **(B)** to explore the difference in MMSE scores between the DEX and saline groups at postoperative day 1.

Considering that the studies included for most of the outcomes were ≤5, this meta-analysis only performed subgroup analyses and publication bias tests for MMSE scores on postoperative day 1. Under the subgroup of cancer types (Figure 4A), the combined results of CRC (WMD [95% CI] = 2.09 [0.85, 3.32], *p* = 0.0009), GC (WMD [95% CI] = 2.63 [1.91, 3.36], *p* < 0.00001), and GIC (WMD [95% CI] = 3.90 [3.36, 4.44], *p* < 0.00001) all suggested that DEX could significantly improve MMSE scores. Moreover, the combined results for three subgroup of cancer types exhibited statistically significant difference (*p* = 0.003). However, there was significant heterogeneity in these subgroups (CRC, *p* < 0.00001; *I*^2^ = 88%; GC, *p* = 0.002, *I*^2^ = 84%; and GIC, not applicable), indicating the cancer type was not a source of heterogeneity in the MMSE score.

In the subgroup of surgery types (Figure 4B), the pooled results of LRS (WMD [95% CI] = 2.56 [1.46, 3.65], *p* < 0.00001) and RS (WMD [95% CI] = 2.50 [1.56, 3.44], *p* < 0.00001) both suggested a significant improvement in MMSE scores in the DEX group compared to the use of saline. However, the pooled data exhibited significant diversity between LRS and RS (*p* = 0.94). Furthermore, significant heterogeneity in these subgroups was observed (LRS, *p* < 0.00001, *I*^2^ = 91%; and RS, *p* < 0.00001, *I*^2^ = 88%), indicating that surgery type was also not a source of heterogeneity in the MMSE score.

In addition, Egger’s test indicated no publication bias among the included studies (*p* = 0.447; Supplementary Figure 2).

### 3.5 Sensitive analysis

The one-by-one elimination method revealed that for outcome indicators, except POCD at postoperative day 1, excluding any single study did not significantly alter the combined results, and the remaining studies continued to be statistically significant and consistent with the original results (Table 2 and Supplementary Figures 3, 4), which further suggested the robust outcomes and stability of this meta-analysis.

**Table 2:** Outcomes of the sensitivity analysis.

Outcomes	No. of studies	Sensitivity analysis	
		WMD/RRs (95% CI)	Robust
MMSE			
Post 1 day	9	2.35 (1.70, 2.99) to 2.69 (2.03, 3.36)	Yes
Post 2 days	5	2.11 (0.35, 3.87) to 3.15 (2.18, 4.13)	Yes
Post 3 days	5	1.53 (1.00, 2.06) to 2.58 (1.16, 4.00)	Yes
Post 7 days	4	1.24 (0.77, 1.71) to 2.15 (0.82, 3.48)	Yes
POCD			
Post 1 day	4	0.31 (0.15, 0.61) to 0.37 (0.22, 0.64)	Yes
Post 7 days	3	0.28 (0.09, 0.84) to 0.67 (0.26, 1.73)	No

WMD, weighted mean difference; RR, risk ratio; CI, confidence interval; MMSE, mini-mental state examination; POCD, postoperative cognitive dysfunction.

## 4 Discussion

This meta-analysis comprehensively probed the effects of DEX on POCD in patients with GICs undergoing RS. The results suggested that the use of DEX improved the postoperative MMSE score and reduced the incidence of POCD compared to the group treated with saline. However, the effect value of the MMSE score gradually decreased in the postoperative period from days 1–7 and the POCD incidence was less stable at postoperative day 7. Impaired cognitive function during the postoperative period may manifest as deficits in daily behavioral capabilities, including a diminished ability to adhere to medical instructions, challenges in self-care, and compromised decision-making capacity. Such behavioral impairments can not only prolong recovery but also elevate the risk of adverse events, such as falls and non-compliance with treatment (9, 40). By enhancing MMSE scores and reducing the incidence of POCD, DEX may help mitigate these risks, thereby facilitating patients’ engagement in postoperative rehabilitation and aiding in the restoration of functional independence. Consequently, the protective effect of DEX on cognitive function appears to wane over time, which is consistent with the observations made by Yang et al. (40). They indicated that the cognitive benefits associated with DEX in elderly surgical patients were most pronounced during the early postoperative period, subsequently diminishing over time. However, whether these patients had concomitant GICs remains unknown. Additionally, Xu et al. (41) conducted a relevant meta-analysis to explore the effects of DEX on systemic inflammation and recovery in patients undergoing digestive tract cancer surgery and found that DEX reduced the incidence of POCD at 24 and 72 h postoperatively. While their conclusions support our findings, their study lacked evidence for MMSE outcomes as well as results from subgroup analyses of tumor type and type of surgery. In this meta-analysis, the incidence of POCD and MMSE scores were included as outcome indicators, and subgroup analyses were performed to reveal the effect of DEX on POCD in patients with GICs undergoing RS, with a more comprehensive perspective and more reliable conclusions.

RS is a conventional strategy for the treatment of gastrointestinal tumors because the digestive tract, an important immune organ, is subject to functional deficits, intestinal bacterial translocation, and systemic inflammatory responses during surgery (41). These inflammatory messages from the peripheral immune system are transmitted to the CNS and ultimately received by recipient brain cells, causing neurotoxicity (42). In addition, systemic inflammation may trigger neuroinflammation via circulating exosomes as mediators (43). During this process, inflammatory factors delivered to the brain parenchyma destroy hippocampal neurons, which are responsible for learning and memory, thereby leading to POCD development (44). Hence, the effects of DEX on cognitive function may be linked to an inflammatory response transmitted to the CNS via the gut-brain axis.

An animal-based study indicated that DEX prevents apoptosis and alleviates cognitive dysfunction in rat hippocampal cells by inhibiting the release of inflammatory cytokines (45). In oncologic surgery, DEX may reduce the degree of intraoperative suppression of immune function, including downregulating levels of pro-inflammatory cytokines (such as TNF-*α* and IL-6) (46). In addition, DEX has been found to reduce serum levels of TNF-α, IL-6, and IL-1β in mice (47). In turn, MMSE scores were negatively correlated with serum TNF-α, IL-6, PI3K, and AKT levels (48). Thus, DEX may alleviate the inflammatory response in the CNS and facilitate postoperative cognitive recovery through the PI3K-Akt signaling pathway.

Neurons in the brain are fundamental to cognitive function, and brain damage caused by neuronal apoptosis can contribute to a decline in cognitive function and learning abilities in patients (49). Activated brain mast cells can induce CNS inflammation and POCD by stimulating microglial activation and neuronal apoptosis (50). A study based on an aged POCD mouse model revealed that NF-κB pathway activation leads to neuronal apoptosis and autophagy, causing cognitive dysfunction, while NF-κB pathway inhibitors can reverse neuronal apoptosis induced by brain injury (51). The protective effects of DEX on neurons have been extensively reported. Chen et al., found that DEX alleviates apoptosis and neurological deficits by regulating NOX2-derived oxidative stress (52). DEX may also inhibit neuronal apoptosis through sigma-1 receptor signaling (53). Sun et al. (54) suggested that DEX reduced hippocampal neuronal apoptosis in the brains of mice with Alzheimer’s disease, improving their cognitive function. Therefore, our hypothesis suggests that DEX may slow down neuronal apoptosis through a series of signaling pathways, thereby protecting against brain injury and CNS inflammation and effectively reducing the incidence of postoperative POCD in patients. However, further experimental studies are required to fully elucidate the molecular regulatory mechanisms involved.

Furthermore, preoperative depression and anxiety have been associated with cognitive deficits in patients undergoing surgery for the removal of solid tumors (55). DEX is proposed as an innovative antidepressant candidate, acting through multiple mechanisms to address various pathophysiological aspects of depression. These mechanisms include adjusting the noradrenergic system, managing neuroinflammation and oxidative stress, influencing Brain-Derived Neurotrophic Factor (BDNF) levels, and modulating neurotransmitter systems including glutamate (56). These findings suggest that depression levels or other variables (eg, psychological, emotional, pharmacodynamic effects of chronic medications) may mediate or moderate the improvement in cognitive performance observed with DEX (57, 58). Future research should not only explore the direct effects of DEX on cognition, but also consider how depression levels or other variables might mediate or moderate these effects. The use of DEX in clinical surgery is becoming increasingly popular, as it has been reported to reduce the risk of POCD. Although previous studies have analyzed the effects of DEX on POCD in patients with GICs, their methodological inconsistencies and sample size limitations have resulted in inconclusive outcomes and insufficient evidence (8, 24, 25). By demonstrating the protective effect of DEX on cognitive function, our meta-analysis provides evidence that could inform perioperative management strategies. Medical professionals may consider integrating DEX into anesthesia protocols for patients undergoing RS, especially those at higher risk of POCD. The strengths of this study are as follows: (1) the inclusion criteria were rigorous, excluding studies with loopholes in research design, data accuracy, and completeness of results reporting; (2) all included studies were RCTs but some studies did not describe in detail the random sequence generation method and allocation concealment measures, which may introduce selection bias and implementation bias. The randomization method was not standardized, which may have led to imbalance in baseline characteristics between the two groups, potentially overestimating or underestimating the efficacy of dexmedetomidine. Inadequate blinding may have introduced subjective bias in researchers’ assessment of MMSE scores or determination of POCD; if researchers anticipated dexmedetomidine to be effective, this could have exaggerated the effect size. Nevertheless, sensitivity analyses showed that the results remained stable even after excluding any individual study, thereby alleviating concerns about bias to some extent; (3) the meta-analysis was performed based on the duration of the follow-up time, showing a decrease in the influence of DEX on cognitive function diminished with time; and (4) no significant publication bias was observed among the included studies with high confidence in results.

### 4.1 Limitations

However, this study has some limitations: (1) significant heterogeneity in the combined results of MMSE scores was observed, with neither cancer nor surgery type identified as an influencing factor of heterogeneity in the subgroup analysis. The differences in DEX dosage and administration schedules included in the study may contribute to heterogeneity by influencing the drug’s neuroprotective efficacy. Most studies did not report patients’ baseline cognitive status (e.g., preoperative MMSE scores), and differences in baseline cognitive function may lead to varying responses to DEX among patients and introduce heterogeneity. The diversity of anesthetic techniques used in the studies, including differences in hypnotics, opioids, and adjunctive medications used in combination with DEX, may contribute to heterogeneity in cognitive outcomes through synergistic or antagonistic effects. Due to the lack of detailed reporting of DEX dosage and other data, as well as the absence of baseline cognitive status information in some studies, the current data were insufficient for meta-regression analysis. Future studies should standardize reporting of DEX dosing regimens, baseline cognitive assessments, and anesthetic protocols to facilitate more in-depth heterogeneity analyses. (2) Although our search strategy included international databases without language restrictions, all identified eligible studies were conducted in China. This geographic limitation should be considered when generalizing findings to other populations. In addition, differences in culture, genetics, medical practices, and anaesthesia protocols may affect the generalisability of the study results. For example, differences in perioperative management in China, such as locally adapted enhanced recovery after surgery (ERAS) protocols, genetic polymorphisms related to drug metabolism and inflammatory responses, and anesthetic drug selection, may all affect the efficacy of DEX. Therefore, the findings of this study may be most applicable to populations with similar perioperative contexts (e.g., standardized ERAS protocols, similar anesthetic regimens). The international multicenter RCTs should be conducted to validate DEX’s effects in diverse GIC cohorts, particularly those with different genetic backgrounds and healthcare systems. (3) The number of included studies was inadequate; therefore, additional high-quality RCTs with larger sample sizes are required to further validate the stability and extrapolation of the results. (4) MMSE was a limited screening tool: The MMSE was less sensitive to subtle cognitive changes (mild impairments in executive function and attention) and may struggle to capture the subtle fluctuations in cognitive function in the early post-operative period. The MMSE is significantly influenced by educational attainment and linguistic-cultural background, and since the participants in this study were all Chinese patients, the results may not be representative of assessments conducted in other linguistic-cultural contexts. Future studies should employ a combination of multidimensional cognitive assessment tools (MMSE combined with MoCA, neuropsychological battery tests) and adjust assessment tools based on the educational background and linguistic characteristics of the study population to enhance the accuracy of cognitive function assessment.

## 5 Conclusion

In conclusion, this meta-analysis suggests that DEX has a protective effect on the cognitive function of patients with GICs undergoing RS. Integrating DEX into anesthesia protocols could potentially improve patient safety by reducing the incidence of POCD, thereby contributing to better postoperative outcomes. Medical professionals can use these findings to make informed decisions about anesthesia management tailored to individual patient needs, balancing the benefits of DEX in cognitive preservation with other clinical considerations. However, high-quality, large-scale RCTs are required to provide robust evidence.

## Data Availability

The original contributions presented in the study are included in the article/Supplementary material, further inquiries can be directed to the corresponding author.
