# Supplementary material for: Effect of dexmedetomidine on postoperative cognitive function in patients with gastrointestinal cancer: a meta-analysis of randomized controlled trials
Source: Front Neurol. 2025 Aug 21;16:1605999. doi: 10.3389/fneur.2025.1605999 (PMC12408329; doi:10.3389/fneur.2025.1605999)
Supplement: Supplementary file 1 [file Data_Sheet_1.docx]

Supplementary Material

## Supplementary Figures


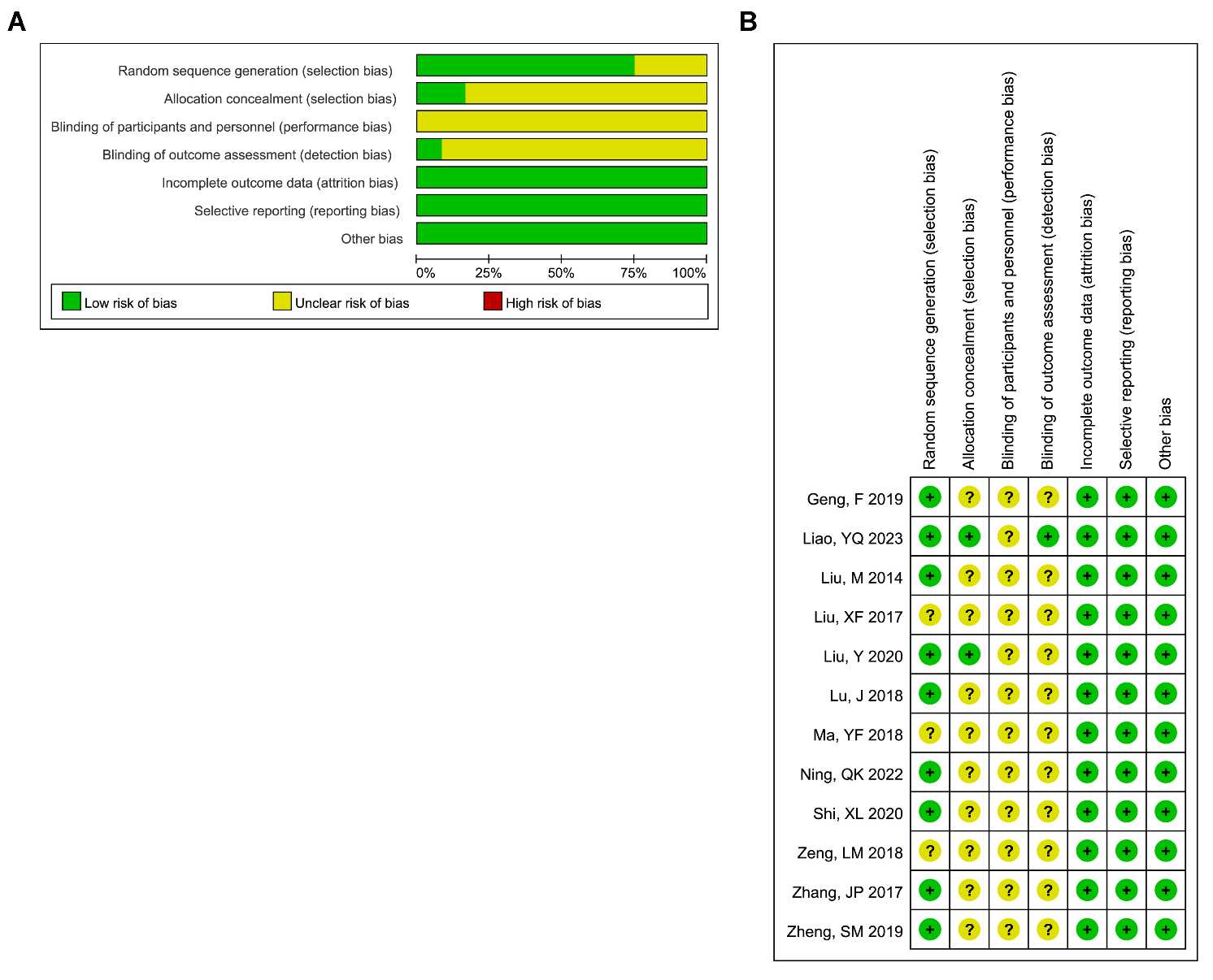


**Supplementary Figure 1. Quality assessment of included studies.**

Assessment of the risk of bias in the included studies. B: Risk of bias for each study.


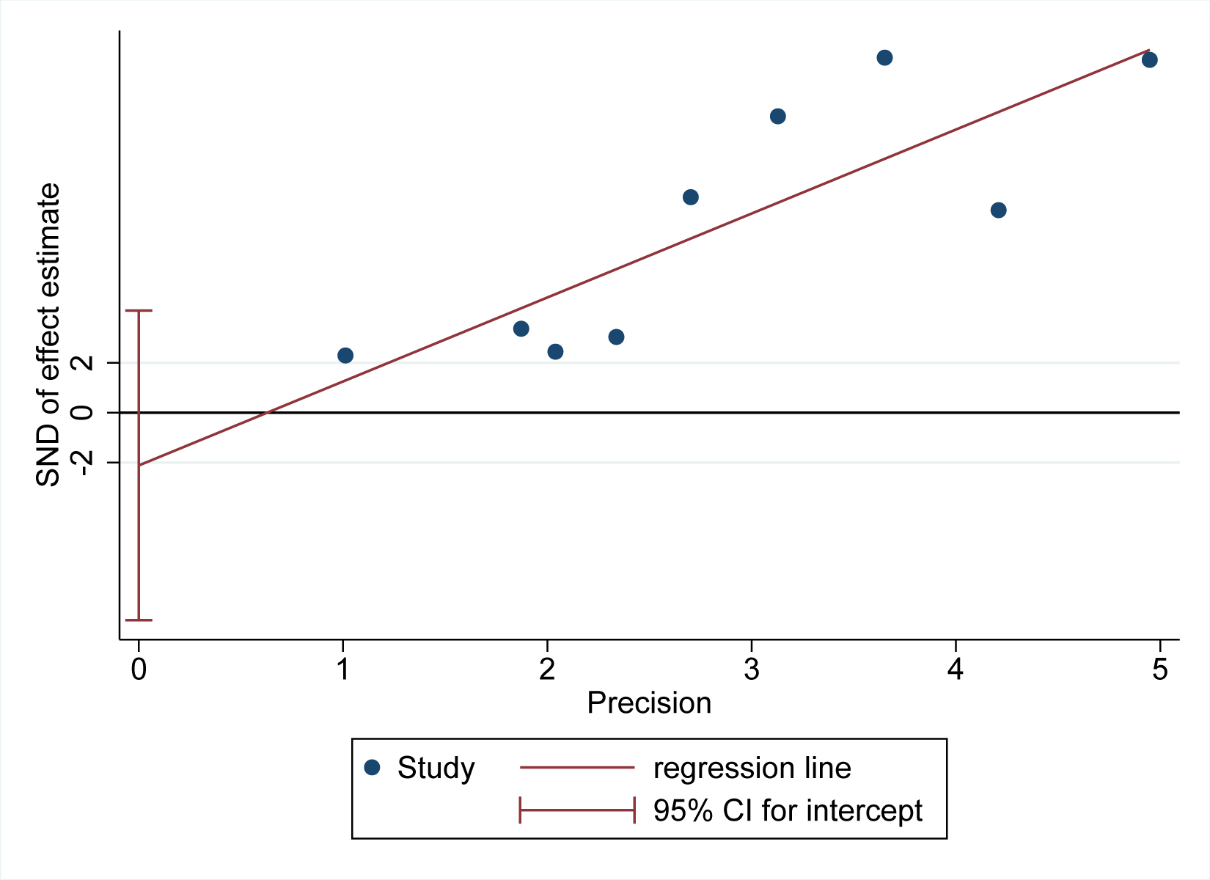


**Supplementary Figure 2. Egger test for assessment of publication bias among included studies**


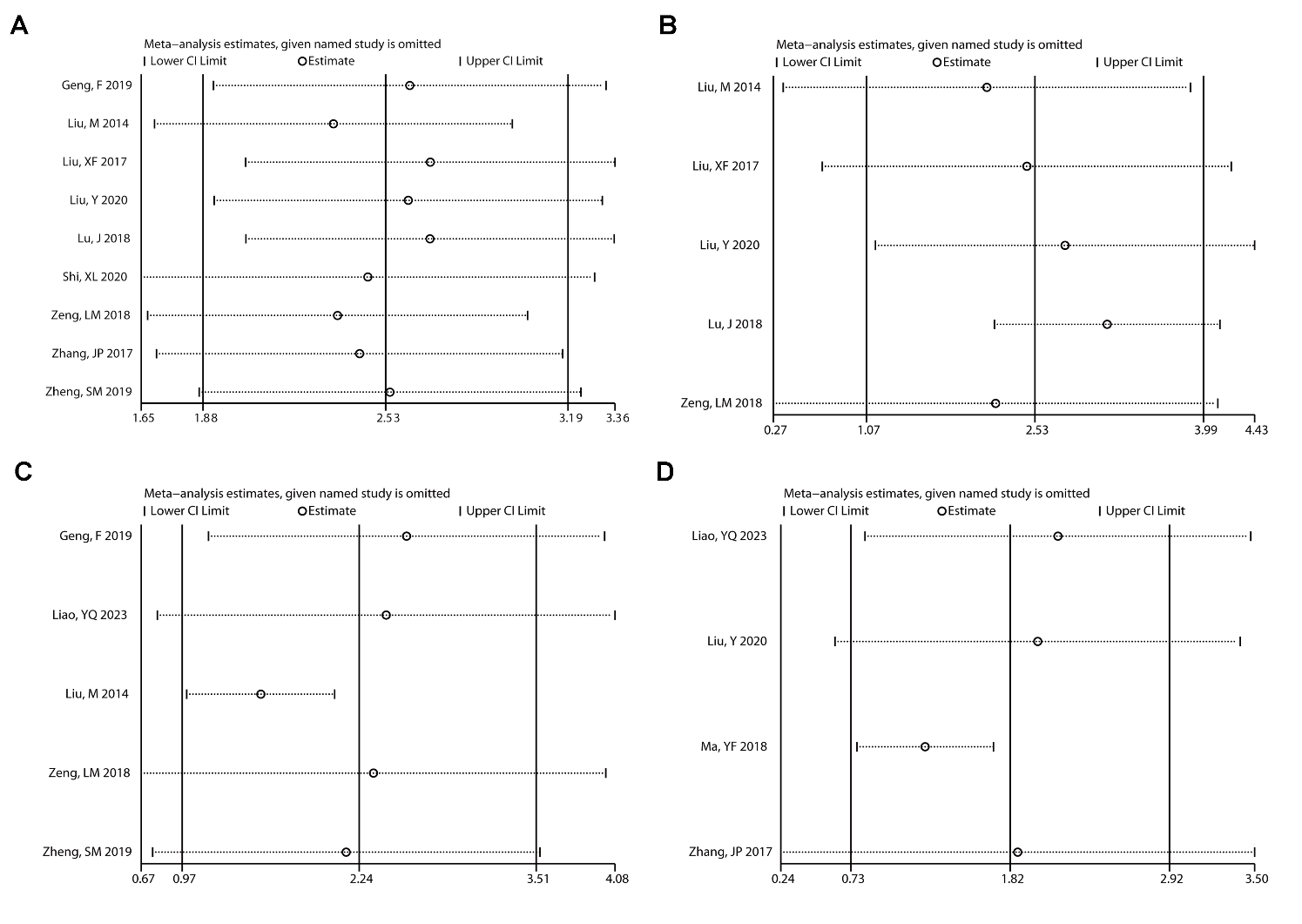


**Supplementary Figure 3** Combined results for MMSE scores at postoperative days 1 (A), 2 (B), 3 (C), and 7 (D) following the one-by-one elimination method.


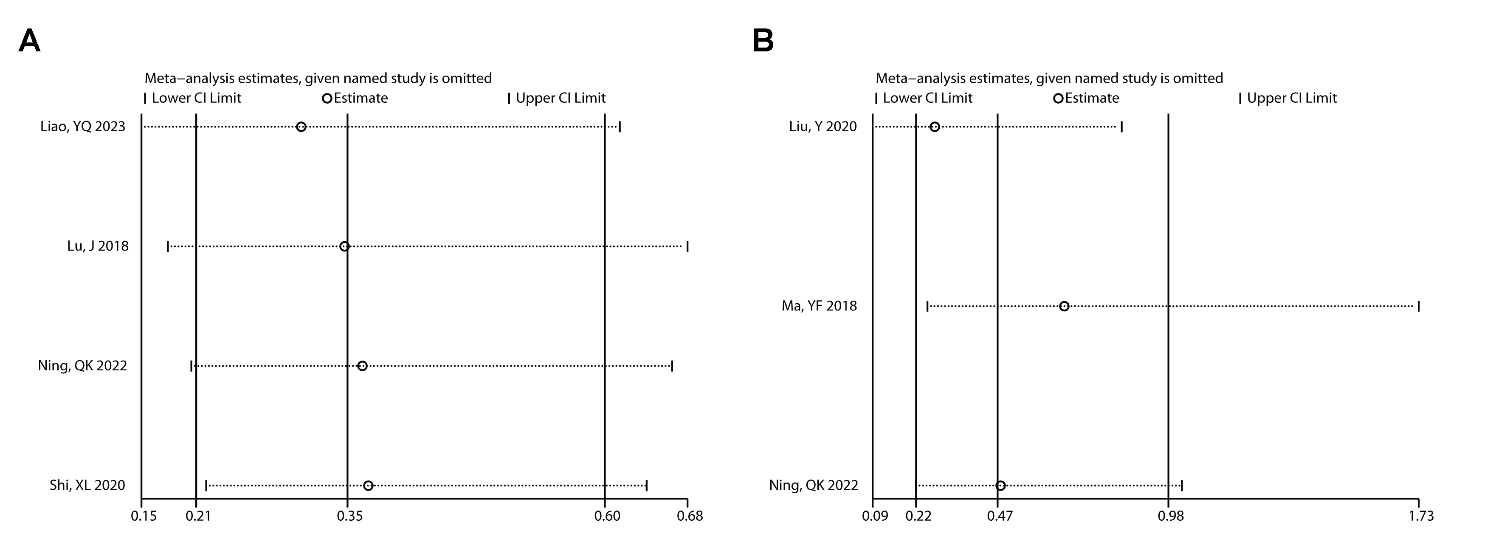


**Supplementary Figure 4** Combined results for POCD incidence at postoperative days 1 (A) and 7 (B) following the one-by-one elimination method.

## Supplementary Tables

**Supplementary Table 1. Search strategies of PubMed**

| Search | Query | Items found |
| --- | --- | --- |
| #1 | "dexmedetomidine"[MeSH Terms] OR "dexmedetomidine"[All Fields] OR "precedex"[All Fields] OR "mpv 1440"[All Fields] | 9233 |
| #2 | "neoplasms"[MeSH Terms] OR "neoplasms"[All Fields] OR "neoplasm"[All Fields] OR "cancer"[All Fields] OR "cancers"[All Fields] OR "cysts"[MeSH Terms] OR "cysts"[All Fields] OR "cyst"[All Fields] OR "tumour"[All Fields] OR "tumor"[All Fields] OR "tumours"[All Fields] OR "tumors"[All Fields] | 5607302 |
| #3 | "cognition"[MeSH Terms] OR "cognition"[All Fields] OR "cognitions"[All Fields] OR "cognitive"[All Fields] OR "cognitively"[All Fields] OR "cognitives"[All Fields] | 749358 |
| #4 | #1 AND #2 AND #3 | 112 |

**Supplementary Table 2. Search strategies of Embase**

| Search | Query | Items found |
| --- | --- | --- |
| #1 | ('dexmedetomidine'/exp OR dexmedetomidine OR precede OR 'mpv 1440'/exp OR 'mpv 1440') | 41671 |
| #2 | ('cancer'/exp OR cancer OR 'tumour'/exp OR tumour OR 'tumor'/exp OR tumor OR 'neoplasm'/exp OR neoplasm OR 'cyst'/exp OR cyst) | 6783258 |
| #3 | Cognitive OR cognition | 725912 |
| #4 | #1 AND #2 AND #3 | 363 |

**Supplementary Table 3. Search strategies of Web of Science**

| Search | Query | Items found |
| --- | --- | --- |
| #1 | Dexmedetomidine OR precede OR "mpv-1440"(All Fields) | 149126 |
| #2 | cancer OR tumour OR tumor OR neoplasm OR cyst (All Fields) | 4488381 |
| #3 | cognitive OR cognition (All Fields) | 701088 |
| #4 | #1 AND #2 AND #3 | 316 |

**Supplementary Table 4. Search strategies of The Cochrane library**

| Search | Query | Items found |
| --- | --- | --- |
| #1 | MeSH descriptor: [Dexmedetomidine] explode all trees | 2639 |
| #2 | (Dexmedetomidine OR precede OR “mpv 1440”):ti,ab,kw (Word variations have been searched) | 21430 |
| #3 | #1 OR #2 | 21430 |
| #4 | MeSH descriptor: [Neoplasms] explode all trees | 113676 |
| #5 | MeSH descriptor: [Cysts] explode all trees | 2920 |
| #6 | (cancer OR tumour OR tumor OR neoplasm OR cyst OR cancers OR tumours OR tumors OR neoplasms OR cysts):ti,ab,kw (Word variations have been searched) | 256182 |
| #7 | #4 OR #5 OR #6 | 269606 |
| #8 | (cognitive OR cognition):ti,ab,kw (Word variations have been searched) | 104657 |
| #9 | #3 AND #7 AND #8 | 129 |

**Supplementary Table 5. The diagnostic criteria and assessment time points for POCD**

| Study | Diagnostic criteria for POCD | Assessment time point (postoperative) |
| --- | --- | --- |
| Geng, F 2019 | MMSE score < 27 | 1d, 3d |
| Liao, YQ 2023 | 1 standard  deviation method of MMSE and MoCA scores | 24h, 72h |
| Liu, M 2014 | MMSE score < 27 | 6h, 1d, 2d, 3d |
| Liu, XF 2017 | MMSE score decreased by ≥2 points compared to preoperative levels | 12h, 24h, 48h |
| Liu, Y 2020 | MMSE score decreased by >2 points compared to preoperative levels | 4h, 12h, 24h, 48h, 7d |
| Lu, J 2018 | MMSE score < 27 | 24h, 48h |
| Ma, YF 2018 | The Z-score method recommended by the International POCD Group | 7d, 1m |
| Ning, QK 2022 | Less than 2 points below the preoperative MMSE score baseline | 1d, 3d, 5d, 7d |
| Shi, XL 2020 | MMSE score < 27 | 24h |
| Zeng, LM 2018 | MMSE score < 27 | 1d, 2d, 3d |
| Zhang, JP 2017 | MMSE score < 27 | 1d, 7d |
| Zheng, SM 2019 | MMSE score < 27 | 1d, 3d |

**Supplementary Table 6. The risk of bias summary table**

| Study | Random method | Allocation Hidden | Blinding participants and researchers | Blinding outcome assessors | Data completeness |
| --- | --- | --- | --- | --- | --- |
| Geng, F 2019 | Random number table method | Not specified | Not specified | Not specified | Complete |
| Liao, YQ 2023 | Random number table method | Yes | Not specified | Not specified | Complete |
| Liu, M 2014 | Random number table method | Not specified | Not specified | Not specified | Complete |
| Liu, XF 2017 | Mentioning randomness, method not specified | Not specified | Not specified | Not specified | Complete |
| Liu, Y 2020 | Random number table method | Not specified | Not specified | Not specified | Complete |
| Lu, J 2018 | Computer random software | Not specified | Not specified | Not specified | Complete |
| Ma, YF 2018 | Mentioning randomness, method not specified | Not specified | Not specified | Not specified | Complete |
| Ning, QK 2022 | Random number table method | Not specified | Not specified | Not specified | Complete |
| Shi, XL 2020 | Mentioning randomness, method not specified | Not specified | Not specified | Not specified | Complete |
| Zeng, LM 2018 | Mentioning randomness, method not specified | Not specified | Not specified | Not specified | Complete |
| Zhang, JP 2017 | Random number table method | Not specified | Not specified | Not specified | Complete |
| Zheng, SM 2019 | Random number table method | Not specified | Not specified | Not specified | Complete |
